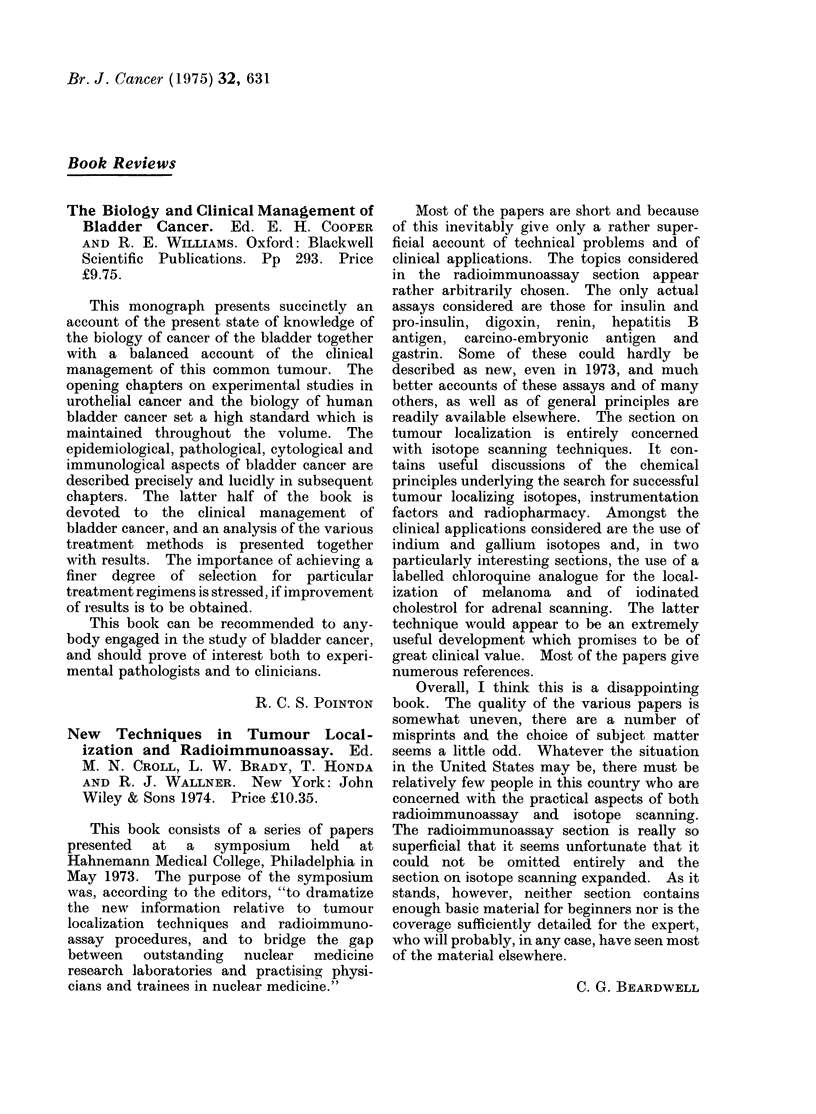# The Biology and Clinical Management of Bladder Cancer

**Published:** 1975-11

**Authors:** R. C. S. Pointon


*Br. J. Cancer* (1975) **32**, 631

### *Book Reviews*

**The Biology and Clinical Management of Bladder Cancer.** Ed. E. H. Cooper and R. E. Williams. Oxford: Blackwell Scientific Publications. Pp 293. Price £9.75.

This monograph presents succinctly an account of the present state of knowledge of the biology of cancer of the bladder together with a balanced account of the clinical management of this common tumour. The opening chapters on experimental studies in urothelial cancer and the biology of human bladder cancer set a high standard which is maintained throughout the volume. The epidemiological, pathological, cytological and immunological aspects of bladder cancer are described precisely and lucidly in subsequent chapters. The latter half of the book is devoted to the clinical management of bladder cancer, and an analysis of the various treatment methods is presented together with results. The importance of achieving a finer degree of selection for particular treatment regimens is stressed, if improvement of results is to be obtained.

This book can be recommended to anybody engaged in the study of bladder cancer, and should prove of interest both to experimental pathologists and to clinicians.

R. C. S. Pointon